# Acetabular cup orientation differs across surgical approaches in primary total hip arthroplasty: a retrospective analysis

**DOI:** 10.1007/s00264-026-06873-5

**Published:** 2026-06-06

**Authors:** Clara Chimeno-Pigrau, Jenaro A. Fernández-Valencia, Alfonso Alías, Adrià Serra, Andrés Combalia, Ernesto Muñoz-Mahamud

**Affiliations:** 1 Departament de Cirurgia i Especialitats Medicoquirúrgiques, Facultat de Medicina i Ciències de la Salut, University of Barcelona, Barcelona, Spain; 2 Department of Orthopaedics and Trauma Surgery, Hospital Clínic de Barcelona, Barcelona, Spain

**Keywords:** Total hip arthroplasty, Acetabular cup positioning, Surgical approach, Cup anteversion, Safe zone

## Abstract

**Purpose:**

Accurate acetabular cup positioning is crucial in primary total hip arthroplasty (THA), as malposition is associated with instability and early failure. We hypothesized that acetabular cup orientation may differ according to the surgical approach used. The aim of this study was to assess whether acetabular anteversion and inclination vary according to surgical approach and whether these differences affect the proportion of acetabular cups positioned outside reference orientation zones.

**Materials and methods:**

A retrospective single-centre comparative study was conducted including 300 primary THAs performed between 2018 and 2022. A stratified random sample of 100 hips per approach was selected: posterolateral (PLA), direct lateral (DLA), and direct anterior (DAA). Cup inclination and anteversion were measured on standardized postoperative radiographs using calibrated digital software. Positioning was analyzed according to the safe zones described by Lewinnek and Reina, as well as the zone corresponding to the lowest observed dislocation ratio reported by Esposito et al. Continuous and categorical variables were compared using appropriate statistical tests (*p* < 0.05).

**Results:**

Mean inclination and anteversion for the overall cohort were 41.5° and 17.6°, respectively. Significant differences were observed between approaches for both inclination (*p* < 0.001) and anteversion (*p* = 0.011), with the DLA demonstrating lower mean anteversion compared with the PLA and DAA. No significant differences were observed in the proportion of cups positioned within the Lewinnek safe zone (*p* = 0.276). However, significant differences were observed in the proportion of cups within the Reina target zone (*p* = 0.0015) and within the zone centred on 48° inclination and 24° anteversion (± 10°) derived from Esposito et al. (*p* = 0.0004).

**Conclusion:**

Acetabular cup positioning appears to vary according to surgical approach in primary THA, particularly regarding anteversion, with the PLA demonstrating higher mean anteversion and the DLA lower values. However, these differences did not translate into clinically relevant differences in positioning within established reference orientation zones according to widely used criteria.

## Introduction

Total hip arthroplasty (THA) is a widely performed and highly successful procedure for the treatment of hip osteoarthritis [1, 2]. Accurate acetabular cup positioning is a critical technical factor, as cup malposition has been associated with instability, impingement, and early failure [3–7]. Consequently, acetabular cup anteversion and inclination remain key parameters in primary THA [8–13].

The surgical approach used in THA may influence the final positioning of the acetabular component. Differences in patient positioning, surgical exposure and intraoperative reference landmarks across approaches can affect the orientation of the acetabular cup [4–17]. However, comparative data evaluating whether acetabular cup anteversion and inclination differ systematically according to the surgical approach remain limited. We hypothesized that acetabular cup positioning differs depending on the surgical approach used in primary THA.

Several “reference orientation zones” for acetabular component orientation have been proposed to reduce the risk of instability, most notably those described by Lewinnek et al. [9] and Reina et al. [18]. Esposito et al. did not identify a statistically significant safe zone; however, their analysis revealed that the zone centered on 48° inclination and 24° anteversion (± 10°) had the lowest observed dislocation ratio (0.70) of all zones evaluated, without reaching statistical significance [19]. However, the relationship between the surgical approach and the likelihood of placing the acetabular cup outside these proposed ranges has not been fully established.

The primary objective of this study was to evaluate whether acetabular cup positioning, specifically anteversion and inclination, differs according to the surgical approach used in primary THA. The secondary objective was to assess the relationship between surgical approach and the proportion of acetabular components positioned outside three predefined reference orientation zones, comparing three different hip approaches.

## Material and methods

### Study design and patient selection

This was a retrospective observational single-centre study. We reviewed all primary total hip arthroplasties performed at our institution between January 2018 and December 2022. From the source cohort, a stratified random sample was created according to surgical approach to obtain comparable groups. One hundred cases were randomly selected for each approach: posterolateral (PLA), direct lateral (DLA) and direct anterior (DAA) performed without traction table, resulting in a total sample of 300 hips. All the surgeries were performed with the aid of fluoroscopic guidance.

Only the index procedure per patient was included; in cases of bilateral THA, the first operated side was analyzed. All procedures were performed by senior hip surgeons with established experience in each specific approach: the PLA was performed by AS and AA, the DAA by JFV, AA and EMM, and the DLA by EMM and AC.

The acetabular components utilized were R3™ and REDAPT™ (Smith & Nephew, Memphis, TN), G7™ (Zimmer Biomet, Warsaw, IN), and Trident™ and X3 RimFit (Stryker Orthopaedics, Mahwah, NJ). The study was approved by the institutional ethics committee (HCB/2024/0822).

### Data collection

Collected variables included age, sex, body mass index, ASA classification, indication for surgery, operated side, surgical approach, acetabular cup size, number of fixation screws, and acetabular cup anteversion and inclination. Postoperative hip dislocations, acute infections and periprosthetic fractures within the first year were recorded.

### Radiographic assessment

**Fig. 1:**
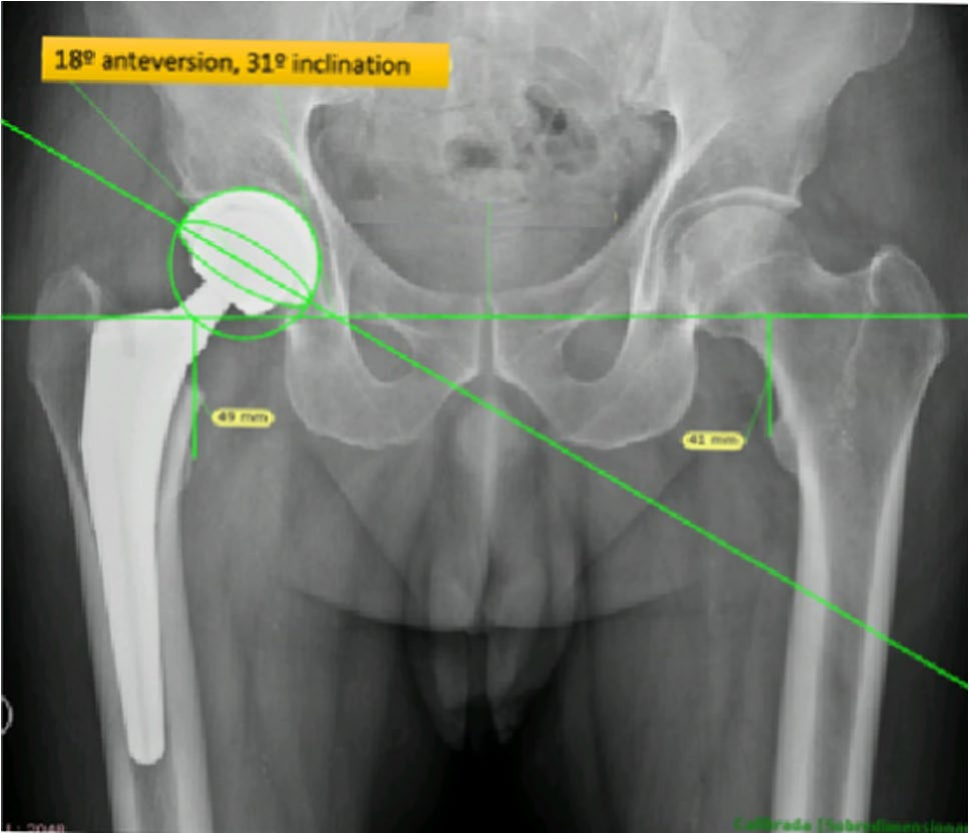
The figure shows representative anteroposterior pelvic radiographs in which acetabular cup anteversion and inclination were measured. In the example shown, the anteversion and inclination are 18° and 31°, respectively

Standardised anteroposterior pelvic radiographs were obtained preoperatively with the patient in the supine position and both hips internally rotated 10–15°. Although a standardized radiographic protocol was used, pelvic tilt and rotation were not formally quantified or controlled for. A dual calibration marker system (KingMark™) was used to correct for magnification. Preoperative templating and postoperative measurements were performed using TraumaCad™ software, which has been validated for the measurement of acetabular cup inclination and version on standard anteroposterior pelvic radiographs (Fig. 1) [20, 21].

**Fig. 2:**
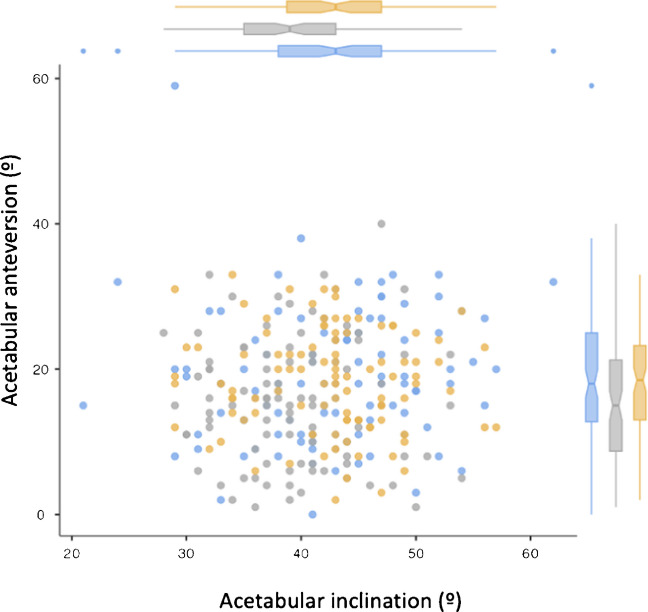
Scatter plot in which each point represents an individual case included in the study, with colors distinguishing the type of hip approach used. The X-axis represents degrees of anteversion, while the Y-axis represents degrees of inclination of the acetabular cup. (Blue: posterolateral approach (PLA); grey: direct lateral approach (DLA); orange: direct anterior approach (DAA))

Postoperative cup inclination was measured as the angle between a horizontal reference line connecting the most inferior points of both ischia and a line drawn along the face of the acetabular component from the medial to the lateral rim. The software automatically calculated the angle between these two lines. Anteversion was assessed using the software’s cup version tool based on the projected ellipse of the acetabular component on the anteroposterior radiograph. A line was drawn along the major axis of the elliptical projection of the cup, and the version tool was adjusted to match the cup margins, allowing automatic calculation of radiographic anteversion according to the ellipse method (Fig. 2). All measurements were performed by a single independent observer (CCP) who was blinded to the surgical approach. Intra- or interobserver reliability was not formally assessed.

Acetabular cup positioning was evaluated according to established reference zones described in the literature. The Lewinnek safe zone was defined as an inclination of 30–50° and an anteversion of 5–25° [9]. The Reina target zone was defined as an inclination of 40–50° and an anteversion of 15–30° [18]. Cups positioned outside these ranges were classified as outliers. Additionally, we applied the zone centered on 48° inclination and 24° anteversion (± 10° for each parameter), corresponding to the region identified by Esposito et al. as having the lowest observed dislocation ratio (0.70) across all zones evaluated in their large prospective cohort, despite the absence of a statistically significant safe zone in that study [22]. This zone was incorporated as a reference of empirically lower dislocation risk, rather than a formally validated safe zone, yielding boundaries of 38–58° inclination and 14–34° anteversion.

### Statistical analysis

Continuous variables were expressed as mean ± standard deviation and compared using one-way ANOVA or Kruskal–Wallis tests, as appropriate. Categorical variables were reported as counts and percentages and compared using chi-square or Fisher’s exact tests. Normality was assessed using the Shapiro–Wilk test. Statistical significance was set at *p* < 0.05. All analyses were performed using JAMOVI (version 2.5.1.0). Acetabular component positioning was considered within the target range only when both inclination and anteversion criteria were simultaneously fulfilled; failure to meet either parameter resulted in classification outside the target range. To account for potential confounding due to baseline imbalances between groups, multivariable linear regression models were performed with cup inclination and anteversion as dependent variables. Surgical approach was included as the main independent variable, adjusting for age, sex, BMI and acetabular cup size. Regression coefficients were reported.

## Results

### Baseline characteristics

Demographic and clinical characteristics of the study population and comparisons between surgical approaches are summarized in Table 1. The cohort included 300 patients (152 men and 148 women) with a mean age of 65.9 years (SD 12.5). The main indication for surgery was primary osteoarthritis (254 cases, 84.7%). Significant differences between approaches were observed for age, sex distribution, body mass index, number of screws, acetabular cup size and cup orientation (Table 1). No relevant differences were found in surgical indication or laterality.

**Table 1:** Demographic and clinical characteristics of the patients included in the study. Statistically significant *p*-values are presented in bold

	All patients	PLA	DLA	DAA	*p*-values
	(*n* = 300)	(*n* = 100)	(*n* = 100)	(*n* = 100)	
Mean (± SD) age in years	65.9 ± 12.5	68.9 ± 11.9	67.3 ± 11.8	61.5 ± 12.1	** < 0.001**
Gender					**0.005**
Male	152	50	38	61	
Female	148	50	62	39	
Mean (± SD) BMI in kg/m^2^	27.6 ± 4.82	28.3 ± 4.6	27.9 ± 5.5	26.7 ± 4	**0.037**
ASA (%)					0.190
I-II	239	74	79	86	
III-IV	61	26	21	14	
Index Surgery (%*)					0.327
Osteoarthritis	254 (85%)	89	83	82	
AVN	26 (9%)	8	7	11	
Dysplasia	15 (5%)	1	8	6	
Hip fracture	5 (2%)	2	2	1	
Laterality (%)					0.187
Left	133	49%	37%	47%	
Right	167	51%	63%	53%	
Number of screws					** < 0.001**
0	197	52	55	90	
1	28	3	24	1	
2	61	38	17	6	
3	13	7	3	3	
4	1	0	1	0	
Cup size					** < 0.001**
42–47 mm	28	9	9	10	
48–53 mm	151	50	67	34	
54–58 mm	111	34	23	54	
59–63 mm	10	7	1	2	
Dislocation (%)	1	1	0	0	NA
Cup orientation					
Mean (± SD) anteversion	17.6 ± 8.36	18.9 ± 9.1	15.5 ± 8.4	18.4 ± 7	**0.01**
Mean (± SD) inclination	41.5 ± 6.76	42.7 ± 7.6	39.4 ± 5.8	42.4 ± 6.3	** < 0.001**

*PLA* Posterolateral Approach, *DLA* Direct Lateral Approach, *DAA* Direct Anterior Approach, *SD* Standard Deviation, *BMI* Body Mass Index), *ASA* American Society of Anesthesiologist, *AVN* Avascular Necrosis

### Acetabular cup positioning

For the entire cohort, mean acetabular *inclination* was 41.5° (SD 6.76°) and mean anteversion was 17.6° (SD 8.36°) (Fig. 2). When analyzed by surgical approach, significant differences were observed in both inclination and anteversion. Mean inclination and anteversion were 42.7° (SD 7.6°) and 18.9° (SD 9.1°) for the PLA, 39.4° (SD 5.9°) and 15.5° (SD 8.4°) for the DLA, and 42.4° (SD 6.3°) and 18.4° (SD 7.1°) for the DAA. These differences were statistically significant for inclination (*p* < 0.001) and anteversion (*p* = 0.011). In multivariable linear regression analyses adjusted for age, sex, BMI, and cup size, using the PLA as the reference category, the DLA remained independently associated with lower cup anteversion (β = − 3.74°, *p* = 0.002) and lower cup inclination (β = − 3.16°, *p* = 0.001). In contrast, no significant differences were observed between the DAA and PLA regarding either cup anteversion (β = − 0.47°, *p* = 0.695) or cup inclination (β = 0.12°, *p* = 0.900).

### Reference orientation zone analysis

**Fig. 3:**
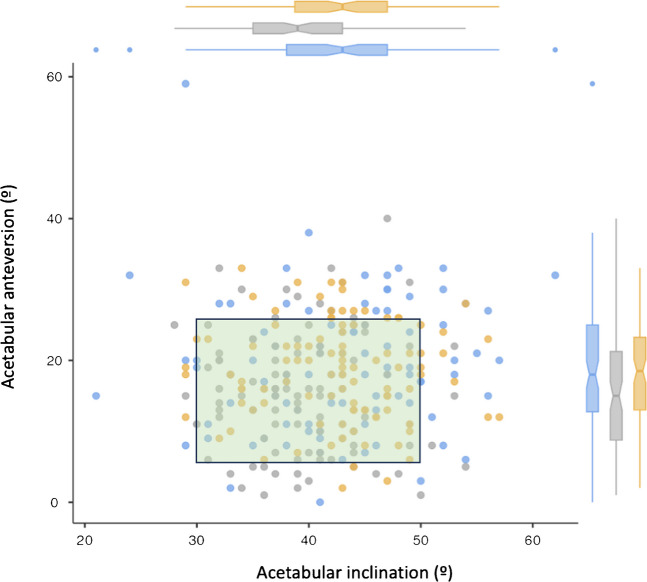
Distribution of cup inclination and anteversion compared to the Lewinnek safe zone (30°–50° inclination and 5°–25° anteversion). Discontinuous circles show mean inclination and anteversion for each approach (Blue: posterolateral approach (PLA); grey: direct lateral approach (DLA); orange: direct anterior approach (DAA))

**Fig. 4:**
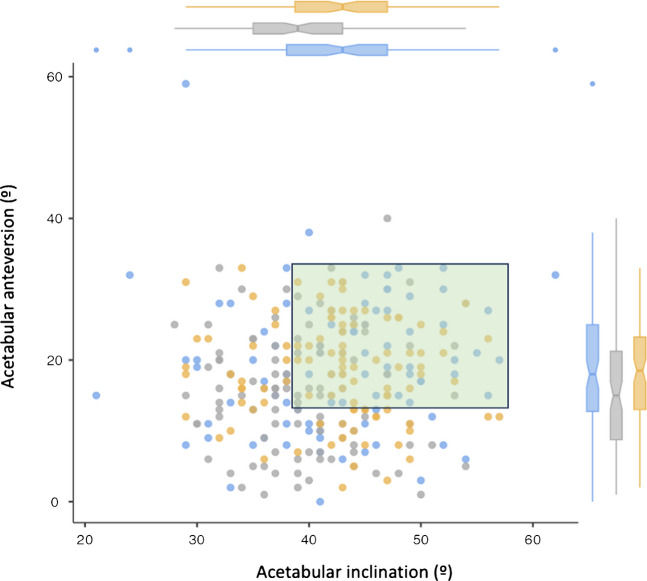
Cup positioning evaluated using the Reina et al. safe zone (40°–50° inclination and 15°–30° anteversion). Discontinuous circles show mean inclination and anteversion for each approach (Blue: posterolateral approach (PLA); grey: direct lateral approach (DLA); orange: direct anterior approach (DAA))

**Fig. 5:**
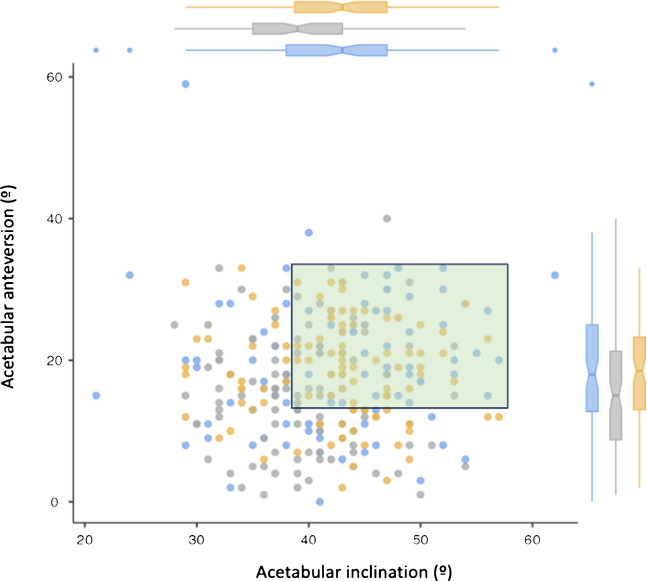
Cup placement compared to the Esposito et al. “lower risk zone” (38°–58° inclination and 14°–34° anteversion). Discontinuous circles show mean inclination and anteversion for each approach (Blue: posterolateral approach (PLA); grey: direct lateral approach (DLA); orange: direct anterior approach (DAA))

Acetabular component positioning was subsequently analyzed according to established reference orientation zones. When the Lewinnek safe zone (30–50° inclination and 5–25° anteversion) was applied, no statistically significant association was observed between surgical approach and the proportion of cups positioned within the target range (*p* = 0.276). The proportion of cups within the Lewinnek zone was 62% for the posterolateral approach (PLA), 72% for the direct lateral approach (DLA), and 70% for the direct anterior approach (DAA) (Fig. 3). In contrast, when the Reina target zone (40–50° inclination and 15–30° anteversion) was applied, differences between approaches were observed (*p* = 0.0015), with 34% of cups within this range for PLA, 18% for DLA, and 41% for DAA (Fig. 4). Similarly, when applying the zone centred on 48° inclination and 24° anteversion (± 10°) derived from Esposito et al. (38–58° inclination and 14–34° anteversion), (Fig. 5) differences between approaches were also observed (*p* = 0.0004), with 55% of cups within this range in both the PLA and DAA groups, compared with 31% in the DLA group.

### Postoperative complications

Postoperative complications were infrequent. One patient in the PLA group experienced a prosthetic dislocation within the first postoperative year, which was successfully treated with closed reduction without recurrence. This patient’s cup position was in 44º of inclination and 10º of anteversion; therefore, it was within Lewinnek’s safe zone but not in Reina’s target zone or the Esposito-derived reference zone. Two cases of deep infection were recorded: one in a 77-year-old male operated via the DLA approach for osteoarthritis and one in a 54-year-old male with femoral head necrosis treated via the DAA approach. Additionally, one periprosthetic acetabular fracture occurred in a 71-year-old patient in the DLA group, requiring revision of the acetabular component. No further major complications were observed.

## Discussion

The most important finding of this study is that acetabular cup positioning differs according to the surgical approach used in primary total hip arthroplasty. Specifically, significant differences were observed in both cup inclination and anteversion across approaches, confirming our hypothesis that the surgical approach influences the final orientation of the acetabular component. In our series, the DLA was associated with lower mean anteversion and inclination compared with both the PLA and the DAA. These findings are consistent with previous reports suggesting that differences in patient positioning, surgical exposure and intraoperative reference landmarks inherent to each approach may affect cup orientation. Chen et al. [13] reported higher anteversion values with the DAA compared with the DLA, while Thompson et al. [16] identified significant differences in cup orientation across multiple approaches. Similarly, Callanan et al. [23] identified surgical approach as the only factor independently associated with cup malposition, whereas Li et al. [24] found lower anteversion values with the DLA compared with the PLA. The potential influence of surgical approach on cup orientation has also been evaluated in robot-assisted THA, with conflicting results. While Kunze et al. [25] reported persistent approach-related differences despite robotic assistance, Hayashi et al. [26] found the anterior approach to be independently associated with less accurate cup positioning. Overall, these findings suggest that although modern technologies may improve accuracy, surgical approach continues to influence acetabular cup orientation [27].

However, although statistically significant differences in inclination and anteversion were observed, these did not translate into differences in the proportion of cups positioned within the Lewinnek safe zone, nor into clinically relevant differences in early postoperative complications. This is further supported by the multivariable analysis, in which the observed differences remained statistically significant after adjustment but were of relatively small magnitude. Therefore, the clinical impact of these variations in cup orientation remains uncertain. These findings highlight the need to distinguish between statistical significance and clinical relevance, as small differences in mean cup orientation may not necessarily result in meaningful differences in patient outcomes.

When acetabular cup orientation was evaluated according to established reference orientation zones, no statistically significant differences between surgical approaches were observed using either the Lewinnek or the Reina criteria. Multiple reference zones were used to provide a comprehensive assessment of cup positioning. The Lewinnek safe zone was considered the primary reference due to its widespread clinical use, whereas the Reina target zone and the Esposito-derived reference zone were included as complementary frameworks to explore alternative definitions of optimal cup orientation. These findings are consistent with those of Kruse et al. [14], who also identified significant differences in cup inclination and anteversion between approaches. Similarly, Abdel et al. [28] reported that cups implanted through a posterior approach were more likely to lie within the Lewinnek safe zone than those implanted through an anterolateral approach, despite the posterior approach being associated with a higher overall risk of dislocation. Reina et al. [18] also observed greater cup anteversion with anterior compared with posterior approaches, further supporting the influence of surgical approach on component orientation. Although differences between approaches were observed when applying more restrictive or alternative reference zones, the absence of differences within the Lewinnek safe zone reinforces the limited clinical impact of these findings.

The low rate of postoperative complications in this study precludes meaningful conclusions regarding the clinical impact of approach-related differences. Only one dislocation was observed, and the overall number of adverse events was small. Therefore, our study was not designed to assess the relationship between cup orientation and clinical outcomes, but rather to describe differences in positioning associated with the surgical approach [29, 30].

Several limitations should be acknowledged. The retrospective design may introduce selection bias, and all radiographic measurements were performed by a single blinded observer, while intra- and interobserver reliability were not formally assessed. In addition, the choice of surgical approach was surgeon-dependent. Although all surgeons had established experience with their respective approaches, experience with the DAA was relatively shorter compared with the other approaches, which may reflect differences in the maturity of the learning curve. Although a stratified random sampling strategy was used to ensure balanced group sizes, the cohorts were not matched for baseline characteristics such as age, sex, body mass index, implant-related variables, spinal pathology or prior spinal arthrodesis. Because surgical approach was not randomly assigned and may have been influenced by patient anatomy, surgeon preference or implant-related considerations, residual confounding cannot be excluded. Furthermore, pelvic tilt and rotation, which may affect the accuracy of radiographic measurements, were not formally assessed. Finally, measurements were based on standard anteroposterior radiographs rather than three-dimensional imaging.

In conclusion, acetabular cup positioning in primary THA appears to vary according to the surgical approach, with the DLA resulting in lower anteversion compared with the PLA and the DAA. However, these differences did not translate into differences in reference orientation zone positioning or early complications. Therefore, although surgical approach may influence cup orientation, our findings do not support the superiority of any specific approach, but rather emphasize the importance of consistent technique and awareness of approach-specific tendencies.

## Data Availability

No datasets were generated or analysed during the current study.

## Declarations

### Statistical analysis and figures

Statistical analyses and graphical representations were performed using JAMOVI software (version 2.5.1.0).
